# A novel wire-guided prefenestration technique for patients with type B aortic dissection: A case report

**DOI:** 10.1097/MD.0000000000036215

**Published:** 2024-01-05

**Authors:** Shuxiong Ge, Zhongyou Xu

**Affiliations:** a Department of Vascular Surgery, People’s Hospital affiliated to Ningbo University, Ningbo, Zhejiang, China.

**Keywords:** left subclavian artery reconstruction, prefesnstion, type B aortic dissection

## Abstract

**Rationale:**

Acute type B aortic dissection (ABAD) is a fatal and severe cardiovascular disease. There are various strategies for dissection involving the left subclavian artery, but limited by the variety and cost of stents, the treatment brings certain obstacles. The aim of this study is to evaluate the effectiveness and safety of the wire-guided prefenestration technique for treating left subclavian artery involvement in patients with arterial dissection.

**Patient concerns:**

A 48-year-old man was transferred to our hospital due to persistent chest and back pain that had lasted for 6 hours.

**Diagnoses:**

Preoperative computed tomography angiogram (CTA) of the thoracic and abdominal aorta diagnosed with ABAD that affected his left subclavian artery, who needed emergency endovascular treatment due to malperfusion of the lower limb arteries.

**Interventions:**

To perform the procedure, a guide wire was inserted through the left brachial artery, exited through the right femoral artery, and then entered the pre-fenestrated hole leading to the main stent. The stent was released while the guide wire was in position, and the left subclavian artery was reconstructed using viabahn.

**Outcomes:**

Thoracic endovascular aortic repair was successfully completed for ABAD. A follow-up CT angiogram of the thoracic and abdominal aorta revealed positive vascular remodeling and no signs of significant internal leakage after one month.

**Lessons:**

This innovative approach offers a secure and efficient treatment option for aortic dissection in individuals who have undergone left subclavian artery reconstruction.

## 1. Introduction

Acute type B aortic dissection (ABAD) is a severe cardiovascular disease that carries a high risk of morbidity and mortality.^[1]^ In fact, once the dissection ruptures, the mortality rate is nearly 100%. The primary symptom of aortic dissection is persistent chest and back pain. However, malperfusion symptoms may also occur, such as abdominal pain, abnormal kidney function, and numbness in the lower extremities, when the blood flow to the superior mesenteric artery, renal arteries, and lower extremities is affected by the aortic dissection.^[2]^ Recently, thoracic endovascular aortic repair (TEVAR) has emerged as the preferred treatment for type B aortic dissection. However, left subclavian artery revascularization presents a challenge due to the insufficient landing zone. One method of revascularization is fenestration, which offers the advantage of avoiding internal leakage.^[3]^ However, this procedure requires skillful operators with sophisticated experience, as well as precise measurements prior to operation. The success rate of fenestration has been a subject of debate among scholars for a long time. In order to improve the success rate of revascularization, we have adopted a novel wire-guided fenestration technique. The new technique is detailed as follows.

## 2. Case report

**Figure 1.**
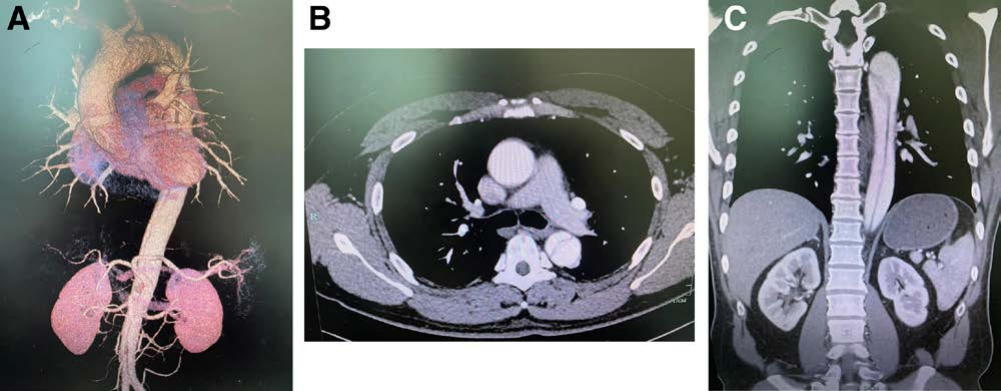
The CTA shows a type B aortic dissection with different anatomical features. (A) Three-dimensional image, (B) transverse section, and (C) coronal section.

A 48-year-old man was transferred to our hospital due to persistent chest and back pain that had lasted for 6 hours. The patient had a history of hypertension, smoking, and alcohol consumption. Upon examination with contrast-enhanced computed tomography angiography, it was discovered that he had thoracic and abdominal aortic dissection (Figure 1). Suddenly, the patient has developed paralysis in their left lower limb, and the blood flow to their left iliac arteries has decreased. As a result, urgent action is required to improve blood supply to the lower extremities.

**Figure 2.**
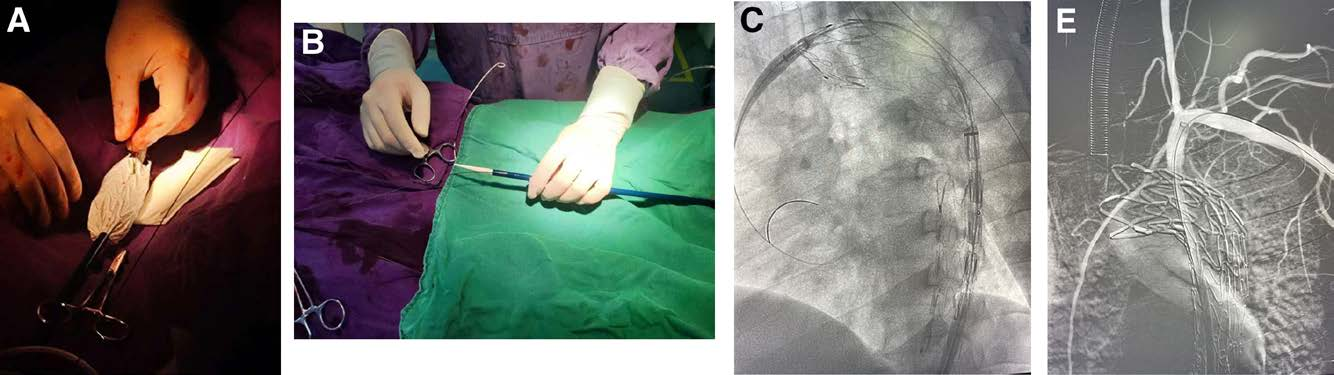
Wire guided prefenestration procedure of Type B aortic dissection. (A) The guide wire from the left subclavian artery enters into the stent from outside of the stent. (B) The position of guide wire after reassembly of the stent. (C) Aortic coated stent released in the distal to the left common carotid artery. (D) The long sheath enters the left subclavian artery. (E) Postoperative aortic angiography.

**Figure 3.**
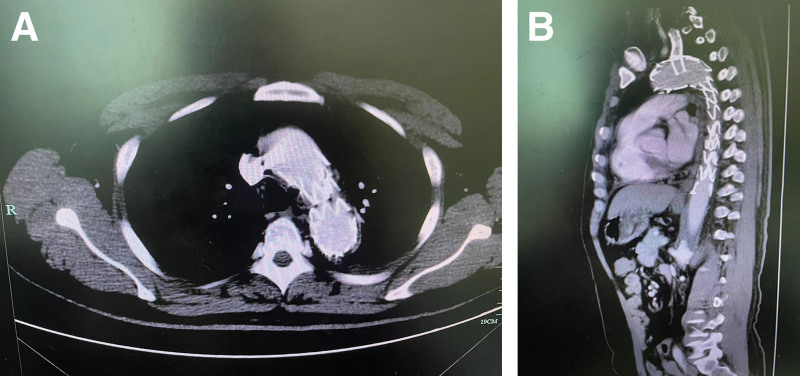
CTA image after endovascular isolation of aortic dissection by covered stent within 1 month. (A) transverse section and (B) sagittal section.

After administering general anesthesia, a 5-Fr sheath was inserted through both the right common femoral artery and left brachial artery. Arteriography revealed that the entry point of the dissection was located within 2cm of the left subclavian artery, tearing in a backward direction towards the left subclavian root and in a forward direction towards the left iliac artery. After administering 5000 units of heparin systemically, a 0.035 inch guide wire was inserted through the left subclavian artery and then retrieved using a snare from the right femoral artery. The fenestration was artificially modified to be 20 mm distal from the proximal markers of the covered stent graft (38–30*200mm, Ankura, China). The size of the scallop was made to match that of the left subclavian artery using eye cautery (Figure 2). Next, the guide wire was inserted through the left brachial artery and entered the stent body from outside of the fenestration hole. The stent-graft was then reloaded into the introducer system simultaneously. The guide wire in the left brachial artery was maintained within the stent when the stent graft system was successfully inserted into the thoracic aorta. The alignment of the opening position of the fenestration with the entry of the left subclavian artery was carefully verified before the stent graft was precisely released. A path was established from the right common femoral artery to the LCCA using a long sheath and wire. A viabahn (11*50mm, Gore, America) was then delivered to the opening of the left subclavian artery, entering the thoracic aorta 0.5cm and deployed. Ascending aortic angiography revealed that there was continuous blood flow in the brachiocephalic trunk, left common carotid artery, and left subclavian artery, with no observed internal leakage in the stent. A follow-up CT angiogram of the thoracic and abdominal aorta revealed positive vascular remodeling and no signs of significant internal leakage after 1 month (Figure 3). The study was conducted with the informed consent of the hospital ethics committee and the patient. The patient consented to the publication of the case.

## 3. Discussion

Thoracic endovascular aortic repair has revolutionized the treatment of aortic dissection by providing a minimally invasive alternative to traditional open surgery. This has resulted in a significant decrease in mortality rates and an increase in survival time for patients with aortic dissection.^[4]^ However, type B aortic dissection is a complex disease that frequently affects the left subclavian artery, presenting unique challenges for endovascular treatment.

Various approaches are currently utilized to reconstruct the flow of the left subclavian artery in order to guarantee adequate blood supply to the brain. These approaches include chimney technique, and fenestration, among others.^[5]^ The chimney technique has been found to increase the risk of type I endoleak, which is caused by a gap between 2 parallel stents.^[6]^ On the other hand, in situ fenestration presents a different challenge, as it is difficult to predict the exact position of the fenestration hole in vivo. This unpredictability can result in a poorly shaped hole, which can destabilize the main stent structure and negatively impact the long-term patency rate of the branch stents.^[7]^ In order to achieve successful vitro fenestration, it is crucial to ensure precise placement and proper sizing of the fenestration holes to guarantee proper alignment. However, 1 major downside is that modifying the stent during surgery can lead to a higher risk of infection and a greater likelihood of postoperative type III endoleak.^[8]^

In this case study, we employed a novel prefenestration technique to reconstruct the left subclavian artery of a patient with aortic dissection. Our approach involved creating a small prefenestration within the aortic coated stent and guiding a wire through the stent from the left brachial artery, circumventing the need for external fenestration. We found that wire-guided prefenestration technology was a simpler and more accessible alternative to traditional methods. Unlike in situ fenestration, this new method reduces the risk of aortic injury. This surgical procedure offers multiple benefits, including reduced operation time, improved fenestration accuracy, and proper blood supply to the brain. Furthermore, the use of the left brachial artery as a puncture point allows for a minimally invasive approach and avoids the need for large surgical incisions.

## 4. Conclusion

This innovative approach provides a secure and efficient solution for aortic dissection in patients who have undergone left subclavian artery reconstruction. Additionally, it proves beneficial for physicians who have recently performed endovascular treatment on complex aortic dissection cases.

## Author contributions

**Supervision:** Zhongyou Xu.

**Validation:** Zhongyou Xu.

**Writing – original draft:** Shuxiong Ge.

**Writing – review & editing:** Shuxiong Ge.
